# Bis[2-(2*H*-benzotriazol-2-yl)-4-methyl-6-(phenyl­imino­methyl-κ*N*)phenolato-κ*O*]palladium(II)

**DOI:** 10.1107/S1600536811049555

**Published:** 2011-11-25

**Authors:** Jing-Kai Su, Chen-Yu Li, Bao-Tsan Ko

**Affiliations:** a Department of Chemistry, Chung Yuan Christian University, Chung-Li 320, Taiwan

## Abstract

In the title complex, [Pd(C_20_H_15_N_4_O)_2_], the Pd^II^ atom is tetra­coordinated by two N atoms and two O atoms from two bidentate imine–benzotriazole phenolate ligands, forming a square-planar environment. The asymmetric unit contains two half-mol­ecules in both of which the Pd^II^ atom lies on a centre of symmetry. The average distances between the Pd^II^ atom and the coordinated O and N atoms are 1.9831 (12) and 2.012 (2) Å, respectively.

## Related literature

For background information, see: Brayton *et al.* (2009 ▶); Li *et al.* (2010 ▶). For related structures, see: Tsai *et al.* (2009 ▶); Lin *et al.* (2010 ▶).
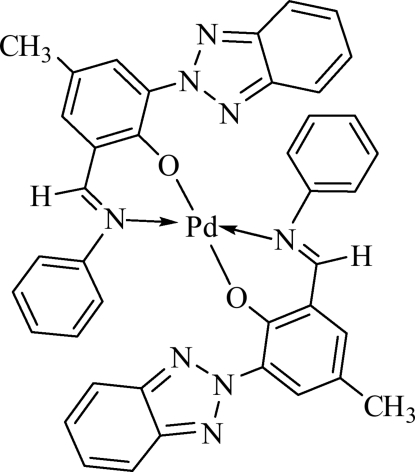


## Experimental

### 

#### Crystal data

[Pd(C_20_H_15_N_4_O)_2_]
*M* *_r_* = 761.12
Triclinic, 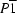

*a* = 11.7509 (2) Å
*b* = 11.9846 (2) Å
*c* = 13.4898 (2) Å
α = 78.808 (1)°
β = 89.357 (1)°
γ = 63.535 (1)°
*V* = 1662.20 (5) Å^3^
*Z* = 2
Mo *K*α radiation
μ = 0.61 mm^−1^
*T* = 296 K
0.20 × 0.12 × 0.08 mm

#### Data collection

Bruker APEXII CCD diffractometer
Absorption correction: multi-scan (*SADABS*; Bruker, 2008 ▶) *T*_min_ = 0.888, *T*_max_ = 0.953
29546 measured reflections
8252 independent reflections
6488 reflections with *I* > 2σ(*I*)
*R*_int_ = 0.023

#### Refinement

*R*[*F* ^2^ > 2σ(*F* ^2^)] = 0.025
*wR*(*F* ^2^) = 0.071
*S* = 1.01
8252 reflections
465 parameters
H-atom parameters constrained
Δρ_max_ = 0.36 e Å^−3^
Δρ_min_ = −0.49 e Å^−3^

### 

Data collection: *APEX2* (Bruker, 2008 ▶); cell refinement: *SAINT-Plus* (Bruker, 2008 ▶); data reduction: *SAINT-Plus*; program(s) used to solve structure: *SHELXS97* (Sheldrick, 2008 ▶); program(s) used to refine structure: *SHELXL97* (Sheldrick, 2008 ▶); molecular graphics: *SHELXTL* (Sheldrick, 2008 ▶); software used to prepare material for publication: *SHELXTL*.

## Supplementary Material

Crystal structure: contains datablock(s) I, global. DOI: 10.1107/S1600536811049555/rk2317sup1.cif

Structure factors: contains datablock(s) I. DOI: 10.1107/S1600536811049555/rk2317Isup2.hkl

Additional supplementary materials:  crystallographic information; 3D view; checkCIF report

## Figures and Tables

**Fig. 1.**
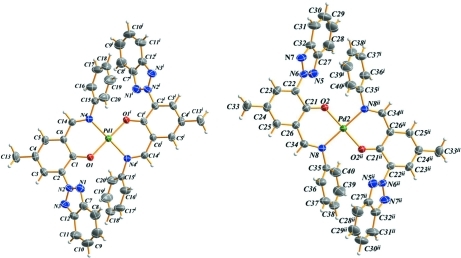
A view of the molecular structure of I with the atom numbering scheme. Displacement ellipsoids are drawn at the 50% probability level. H atoms are presented as a small spheres of arbitrary radius. Symmetry codes: (i) -x+1, -y+1, -z; (ii) -x+2, -y+1, -z+1.

**Fig. 2.**
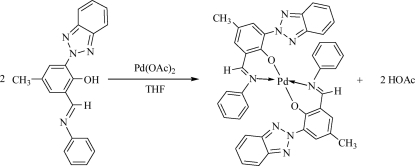
The synthetic procedure of the title compound I.

## supplementary crystallographic information

### Comment

Recently, Brayton *et al.* (2009) reported the synthesis and characterization of the air- and moisture-stable bis(phenoxyketimine) Pd(II) complex, and it was also demonstrated effectively to catalyze Suzuki-Miyaura cross coupling reaction. Experimental results exhibited that it catalyzed the coupling of unactivated aryl bromides with boronic acids in good yields under mild temperature and short reaction time. Therefore, our group is interested in the synthesis and preparation of palladium complexes derived from *N, O*-bidentate ligands. For example, our group has successfully synthesized and structural characterized the Pd(II) complex with methylphenyl-diphenylazo-naphtolate ligands (Lin *et al.*, 2010). Most recently, we also reported the synthesis and crystal structure of the imine group substituted benzotriazole-phenolate derivative (Li *et al.*, 2010). In term of coordination chemistry, the imine-phenolate group can provide the better *N, O*-bidentate chelation to stabilize the transition metal or main group metal complexes. Therefore, we describe the synthesis and crystal structure of *N, O*-bidentate imine-benzotriazole phenolate ligand incorporated Pd^II^ complex, I, a potential catalyst for palladium-catalyzed Suzuki cross-coupling reactions (Scheme 1).

The solid structure of I reveals a monomeric Pd^II^ complex (Fig. 1) including two six-membered rings coordinated from two *N, O*-bidentate imine-benzotriazole phenolate ligands. We found that the asymmetric unit has two half molecules in which both Pd atoms lies on a centre of symmetry. The geometry around each Pd atom is tetra-coordinated with a normal square planar environment in which two nitrogen atoms and two oxygen atoms are coplanar. The two N atoms and two O atoms around Pd atom are *trans* to each other with average bond angle of O1–Pd1–N4 = 90.16 (6)°, O1–Pd1–N4^i^ = 89.84 (6)°, O2–Pd2–N8 = 90.56 (6)°, and O2–Pd2–N8^ii^ = 89.44 (6)°. Symmetry codes: (i) -*x*+1, -*y*+1, -*z*; (ii) -*x*+2, -*y*+1, -*z*+1. The average distances between the Pd atom and O and N (imino nitrogen) are 1.9831 (12)Å and 2.0119 (15)Å, respectively. These bond distances and angles are similar to our earlier reports for the crystal structures of bis[4-methyl-2-(2*H*-benzotriazol-2-yl)phenolato]palladium(II) (Tsai *et al.*, 2009) and bis{(1-[(*E*)-*o*-tolyldiazenyl)naphthalen-2-yloxy]palladium(II) (Lin *et al.*, 2010).

### Experimental

The title complex was synthesized by the following procedures (Fig. 2): (*E*)-2-(2*H*-benzotriazol-2-yl)-4-methyl-6-[(phenylimino)methyl] phenol (0.66 g, 2.0 mmol) and Pd(O*Ac*)_2_ (0.22 g, 1.0 mmol) was stirred at 298 K in *THF* (25 ml) for 24 h. Volatile materials were removed under vacuum and the residue was washed twice from hexane solution to give red solids. The resulting solids were crystallized from CH_2_Cl_2_/Hexane (1:5) solution to yield red crystals.

### Refinement

The H atoms were placed in idealized positions and constrained to ride on their parent atoms, with C–H = 0.93Å with *U_iso_*(H) = 1.2*U*_eq_(C) for phenyl hydrogen; 0.96Å with *U*_iso_(H) = 1.5*U*_eq_(C) for CH_3_ groups.

### Crystal data

**Table:**

[Pd(C_20_H_15_N_4_O)_2_]	*Z* = 2
*M**_r_* = 761.12	*F*(000) = 776
Triclinic, *P*1	*D*_x_ = 1.521 Mg m^−^^3^
Hall symbol: -P 1	Mo *K*α radiation, λ = 0.71073 Å
*a* = 11.7509 (2) Å	Cell parameters from 9762 reflections
*b* = 11.9846 (2) Å	θ = 2.2–28.3°
*c* = 13.4898 (2) Å	µ = 0.61 mm^−^^1^
α = 78.808 (1)°	*T* = 296 K
β = 89.357 (1)°	Block, red
γ = 63.535 (1)°	0.20 × 0.12 × 0.08 mm
*V* = 1662.20 (5) Å^3^	

### Data collection

**Table:**

Bruker APEXII CCD diffractometer	8252 independent reflections
Radiation source: fine-focus sealed tube	6488 reflections with *I* > 2σ(*I*)
graphite	*R*_int_ = 0.023
Detector resolution: 8.3333 pixels mm^-1^	θ_max_ = 28.3°, θ_min_ = 1.5°
φ and ω scans	*h* = −15→15
Absorption correction: multi-scan (*SADABS*; Bruker, 2008)	*k* = −15→15
*T*_min_ = 0.888, *T*_max_ = 0.953	*l* = −17→17
29546 measured reflections	

### Refinement

**Table:**

Refinement on *F*^2^	Primary atom site location: structure-invariant direct methods
Least-squares matrix: full	Secondary atom site location: difference Fourier map
*R*[*F*^2^ > 2σ(*F*^2^)] = 0.025	Hydrogen site location: inferred from neighbouring sites
*wR*(*F*^2^) = 0.071	H-atom parameters constrained
*S* = 1.01	*w* = 1/[σ^2^(*F*_o_^2^) + (0.0283*P*)^2^ + 1.0203*P*] where *P* = (*F*_o_^2^ + 2*F*_c_^2^)/3
8252 reflections	(Δ/σ)_max_ < 0.001
465 parameters	Δρ_max_ = 0.36 e Å^−^^3^
0 restraints	Δρ_min_ = −0.49 e Å^−^^3^

### Special details

**Table:**

Geometry. All s.u.'s (except the s.u. in the dihedral angle between two l.s. planes) are estimated using the full covariance matrix. The cell s.u.'s are taken into account individually in the estimation of s.u.'s in distances, angles and torsion angles; correlations between s.u.'s in cell parameters are only used when they are defined by crystal symmetry. An approximate (isotropic) treatment of cell s.u.'s is used for estimating s.u.'s involving l.s. planes.
Refinement. Refinement of *F*^2^ against ALL reflections. The weighted *R*-factor *wR* and goodness of fit *S* are based on *F*^2^, conventional *R*-factors *R* are based on *F*, with *F* set to zero for negative *F*^2^. The threshold expression of *F*^2^ > σ(*F*^2^) is used only for calculating *R*-factors(gt) *etc*. and is not relevant to the choice of reflections for refinement. *R*-factors based on *F*^2^ are statistically about twice as large as those based on *F*, and *R*-factors based on ALL data will be even larger.

### Fractional atomic coordinates and isotropic or equivalent isotropic displacement parameters (Å^2^)

**Table:**

	*x*	*y*	*z*	*U*_iso_*/*U*_eq_	
Pd1	0.5000	0.5000	0.0000	0.02975 (5)	
O1	0.43565 (12)	0.64409 (13)	0.07104 (10)	0.0382 (3)	
N1	0.47080 (16)	0.81318 (17)	0.17120 (13)	0.0423 (4)	
N2	0.37066 (15)	0.80246 (15)	0.20916 (12)	0.0360 (3)	
N3	0.35846 (18)	0.8031 (2)	0.30772 (13)	0.0505 (5)	
N4	0.34666 (14)	0.59491 (15)	−0.10154 (11)	0.0345 (3)	
C1	0.31532 (16)	0.71915 (17)	0.07416 (13)	0.0327 (4)	
C2	0.27672 (17)	0.79683 (17)	0.14747 (14)	0.0339 (4)	
C3	0.15029 (18)	0.86425 (18)	0.16344 (15)	0.0389 (4)	
H3B	0.1296	0.9107	0.2143	0.047*	
C4	0.05239 (18)	0.86491 (19)	0.10559 (16)	0.0411 (4)	
C5	0.08597 (18)	0.80335 (18)	0.02622 (16)	0.0404 (4)	
H5A	0.0219	0.8094	−0.0175	0.049*	
C6	0.21473 (17)	0.73097 (17)	0.00869 (14)	0.0347 (4)	
C7	0.5317 (2)	0.8193 (2)	0.25227 (16)	0.0446 (5)	
C8	0.6486 (2)	0.8243 (3)	0.2612 (2)	0.0613 (6)	
H8A	0.6944	0.8286	0.2051	0.074*	
C9	0.6915 (3)	0.8228 (3)	0.3538 (2)	0.0715 (8)	
H9A	0.7690	0.8248	0.3618	0.086*	
C10	0.6222 (3)	0.8181 (3)	0.4388 (2)	0.0828 (9)	
H10A	0.6556	0.8167	0.5014	0.099*	
C11	0.5082 (3)	0.8155 (3)	0.4324 (2)	0.0774 (9)	
H11A	0.4621	0.8148	0.4889	0.093*	
C12	0.4625 (2)	0.8140 (2)	0.33610 (17)	0.0509 (5)	
C13	−0.0850 (2)	0.9317 (2)	0.1297 (2)	0.0566 (6)	
H13A	−0.1404	0.9596	0.0688	0.085*	
H13B	−0.0977	1.0040	0.1573	0.085*	
H13C	−0.1040	0.8737	0.1783	0.085*	
C14	0.23845 (17)	0.67832 (18)	−0.08024 (14)	0.0366 (4)	
H14A	0.1691	0.7070	−0.1274	0.044*	
C15	0.35559 (17)	0.56714 (19)	−0.20098 (14)	0.0380 (4)	
C16	0.2814 (2)	0.5200 (2)	−0.23682 (17)	0.0525 (5)	
H16A	0.2227	0.5051	−0.1966	0.063*	
C17	0.2939 (3)	0.4942 (2)	−0.33368 (18)	0.0623 (7)	
H17A	0.2443	0.4612	−0.3579	0.075*	
C18	0.3783 (3)	0.5171 (3)	−0.39271 (18)	0.0696 (8)	
H18A	0.3864	0.4997	−0.4574	0.084*	
C19	0.4516 (3)	0.5657 (4)	−0.3574 (2)	0.0831 (10)	
H19A	0.5088	0.5821	−0.3985	0.100*	
C20	0.4411 (2)	0.5905 (3)	−0.26088 (18)	0.0650 (7)	
H20A	0.4915	0.6228	−0.2367	0.078*	
Pd2	1.0000	0.5000	0.5000	0.02949 (5)	
O2	1.05982 (13)	0.35405 (13)	0.43133 (10)	0.0372 (3)	
N5	1.24404 (16)	0.17309 (17)	0.33049 (13)	0.0438 (4)	
N6	1.13063 (15)	0.19195 (15)	0.29581 (12)	0.0367 (3)	
N7	1.11407 (19)	0.19435 (19)	0.19740 (13)	0.0495 (4)	
N8	0.92774 (14)	0.41267 (15)	0.60629 (11)	0.0342 (3)	
C21	1.00565 (17)	0.27973 (17)	0.43401 (13)	0.0332 (4)	
C22	1.03292 (18)	0.20029 (18)	0.36207 (14)	0.0353 (4)	
C23	0.96764 (19)	0.13146 (18)	0.35389 (15)	0.0404 (4)	
H23A	0.9857	0.0847	0.3032	0.049*	
C24	0.87544 (19)	0.12903 (19)	0.41863 (16)	0.0418 (4)	
C25	0.85671 (19)	0.19365 (18)	0.49572 (16)	0.0398 (4)	
H25A	0.8011	0.1877	0.5437	0.048*	
C26	0.91890 (17)	0.26881 (17)	0.50476 (14)	0.0343 (4)	
C27	1.3086 (2)	0.1642 (2)	0.24674 (16)	0.0459 (5)	
C28	1.4334 (2)	0.1497 (3)	0.2344 (2)	0.0654 (7)	
H28A	1.4873	0.1399	0.2889	0.079*	
C29	1.4707 (3)	0.1509 (3)	0.1390 (3)	0.0804 (9)	
H29A	1.5519	0.1431	0.1276	0.096*	
C30	1.3908 (4)	0.1634 (3)	0.0565 (2)	0.0877 (10)	
H30A	1.4211	0.1632	−0.0075	0.105*	
C31	1.2719 (3)	0.1756 (3)	0.0668 (2)	0.0803 (9)	
H31A	1.2204	0.1829	0.0117	0.096*	
C32	1.2287 (2)	0.1768 (2)	0.16516 (17)	0.0518 (5)	
C33	0.8029 (2)	0.0552 (2)	0.4060 (2)	0.0564 (6)	
H33A	0.7698	0.0361	0.4690	0.085*	
H33B	0.8591	−0.0229	0.3864	0.085*	
H33C	0.7336	0.1053	0.3545	0.085*	
C34	0.89495 (17)	0.32790 (18)	0.59048 (14)	0.0358 (4)	
H34A	0.8509	0.3023	0.6402	0.043*	
C35	0.90674 (19)	0.44738 (18)	0.70374 (14)	0.0370 (4)	
C36	0.7882 (2)	0.4909 (2)	0.73935 (16)	0.0507 (5)	
H36A	0.7188	0.5005	0.6998	0.061*	
C37	0.7719 (3)	0.5207 (2)	0.83463 (18)	0.0600 (6)	
H37A	0.6915	0.5507	0.8588	0.072*	
C38	0.8734 (3)	0.5058 (3)	0.89235 (18)	0.0696 (8)	
H38A	0.8625	0.5256	0.9562	0.084*	
C39	0.9919 (3)	0.4619 (4)	0.8568 (2)	0.0814 (10)	
H39A	1.0613	0.4511	0.8971	0.098*	
C40	1.0090 (3)	0.4336 (3)	0.76148 (18)	0.0616 (7)	
H40A	1.0892	0.4054	0.7369	0.074*	

### Atomic displacement parameters (Å^2^)

**Table:**

	*U*^11^	*U*^22^	*U*^33^	*U*^12^	*U*^13^	*U*^23^
Pd1	0.02420 (9)	0.03341 (10)	0.03039 (10)	−0.00941 (7)	0.00205 (7)	−0.01305 (7)
O1	0.0277 (6)	0.0416 (7)	0.0447 (7)	−0.0098 (5)	0.0018 (5)	−0.0226 (6)
N1	0.0431 (9)	0.0512 (10)	0.0439 (9)	−0.0278 (8)	0.0128 (7)	−0.0188 (8)
N2	0.0375 (8)	0.0429 (9)	0.0327 (8)	−0.0194 (7)	0.0100 (6)	−0.0167 (7)
N3	0.0476 (10)	0.0707 (13)	0.0347 (9)	−0.0239 (9)	0.0111 (7)	−0.0223 (9)
N4	0.0307 (7)	0.0398 (8)	0.0330 (8)	−0.0137 (6)	0.0016 (6)	−0.0130 (6)
C1	0.0310 (8)	0.0316 (9)	0.0351 (9)	−0.0128 (7)	0.0052 (7)	−0.0101 (7)
C2	0.0341 (9)	0.0340 (9)	0.0351 (9)	−0.0155 (7)	0.0057 (7)	−0.0108 (7)
C3	0.0387 (10)	0.0370 (10)	0.0416 (10)	−0.0148 (8)	0.0135 (8)	−0.0161 (8)
C4	0.0328 (9)	0.0371 (10)	0.0505 (11)	−0.0118 (8)	0.0113 (8)	−0.0131 (9)
C5	0.0303 (9)	0.0388 (10)	0.0501 (11)	−0.0125 (8)	0.0027 (8)	−0.0128 (9)
C6	0.0302 (8)	0.0325 (9)	0.0391 (10)	−0.0106 (7)	0.0037 (7)	−0.0115 (7)
C7	0.0454 (11)	0.0463 (11)	0.0491 (12)	−0.0227 (9)	0.0052 (9)	−0.0207 (9)
C8	0.0548 (14)	0.0682 (16)	0.0763 (17)	−0.0369 (13)	0.0044 (12)	−0.0265 (14)
C9	0.0623 (16)	0.0740 (18)	0.092 (2)	−0.0357 (14)	−0.0092 (15)	−0.0337 (16)
C10	0.078 (2)	0.100 (2)	0.0728 (19)	−0.0324 (18)	−0.0202 (16)	−0.0410 (17)
C11	0.0748 (18)	0.110 (2)	0.0485 (14)	−0.0353 (17)	0.0029 (13)	−0.0367 (15)
C12	0.0480 (12)	0.0611 (14)	0.0446 (12)	−0.0199 (11)	0.0031 (9)	−0.0252 (10)
C13	0.0354 (11)	0.0584 (14)	0.0748 (16)	−0.0152 (10)	0.0181 (10)	−0.0275 (12)
C14	0.0309 (9)	0.0383 (10)	0.0386 (10)	−0.0127 (8)	−0.0017 (7)	−0.0109 (8)
C15	0.0323 (9)	0.0419 (10)	0.0341 (9)	−0.0099 (8)	−0.0012 (7)	−0.0123 (8)
C16	0.0625 (14)	0.0633 (14)	0.0415 (11)	−0.0351 (12)	0.0007 (10)	−0.0159 (10)
C17	0.0790 (17)	0.0594 (15)	0.0460 (13)	−0.0261 (13)	−0.0129 (12)	−0.0180 (11)
C18	0.0638 (16)	0.0858 (19)	0.0366 (12)	−0.0092 (14)	−0.0002 (11)	−0.0247 (12)
C19	0.0623 (17)	0.144 (3)	0.0438 (14)	−0.0443 (19)	0.0163 (12)	−0.0287 (17)
C20	0.0539 (14)	0.108 (2)	0.0458 (13)	−0.0435 (15)	0.0106 (10)	−0.0262 (14)
Pd2	0.03257 (10)	0.03457 (10)	0.02779 (9)	−0.01861 (8)	0.00840 (7)	−0.01249 (7)
O2	0.0436 (7)	0.0418 (7)	0.0391 (7)	−0.0264 (6)	0.0165 (6)	−0.0200 (6)
N5	0.0385 (9)	0.0511 (10)	0.0433 (9)	−0.0185 (8)	0.0066 (7)	−0.0179 (8)
N6	0.0413 (8)	0.0410 (9)	0.0319 (8)	−0.0191 (7)	0.0063 (6)	−0.0161 (7)
N7	0.0619 (11)	0.0661 (12)	0.0326 (9)	−0.0350 (10)	0.0095 (8)	−0.0218 (8)
N8	0.0377 (8)	0.0402 (8)	0.0291 (7)	−0.0200 (7)	0.0082 (6)	−0.0117 (6)
C21	0.0342 (9)	0.0323 (9)	0.0332 (9)	−0.0141 (7)	0.0037 (7)	−0.0093 (7)
C22	0.0366 (9)	0.0359 (9)	0.0346 (9)	−0.0154 (8)	0.0058 (7)	−0.0126 (7)
C23	0.0451 (11)	0.0366 (10)	0.0437 (11)	−0.0185 (8)	0.0025 (8)	−0.0175 (8)
C24	0.0419 (10)	0.0364 (10)	0.0516 (12)	−0.0198 (8)	0.0037 (9)	−0.0135 (9)
C25	0.0392 (10)	0.0379 (10)	0.0467 (11)	−0.0201 (8)	0.0092 (8)	−0.0123 (8)
C26	0.0378 (9)	0.0329 (9)	0.0353 (9)	−0.0174 (8)	0.0058 (7)	−0.0103 (7)
C27	0.0474 (11)	0.0448 (11)	0.0479 (12)	−0.0198 (9)	0.0163 (9)	−0.0185 (9)
C28	0.0496 (13)	0.0705 (17)	0.0774 (18)	−0.0254 (12)	0.0197 (12)	−0.0234 (14)
C29	0.0671 (18)	0.081 (2)	0.098 (2)	−0.0346 (16)	0.0458 (17)	−0.0293 (18)
C30	0.103 (3)	0.106 (3)	0.0674 (19)	−0.053 (2)	0.0529 (19)	−0.0356 (18)
C31	0.102 (2)	0.110 (3)	0.0480 (14)	−0.059 (2)	0.0322 (15)	−0.0344 (16)
C32	0.0650 (14)	0.0593 (14)	0.0410 (11)	−0.0323 (12)	0.0183 (10)	−0.0225 (10)
C33	0.0574 (14)	0.0551 (14)	0.0738 (16)	−0.0356 (12)	0.0101 (12)	−0.0261 (12)
C34	0.0372 (9)	0.0390 (10)	0.0344 (9)	−0.0196 (8)	0.0089 (7)	−0.0091 (8)
C35	0.0486 (11)	0.0412 (10)	0.0296 (9)	−0.0265 (9)	0.0082 (8)	−0.0108 (8)
C36	0.0508 (12)	0.0641 (14)	0.0388 (11)	−0.0247 (11)	0.0115 (9)	−0.0180 (10)
C37	0.0767 (17)	0.0624 (15)	0.0436 (12)	−0.0306 (13)	0.0264 (12)	−0.0204 (11)
C38	0.119 (2)	0.0822 (19)	0.0357 (12)	−0.0657 (19)	0.0193 (14)	−0.0243 (12)
C39	0.104 (2)	0.133 (3)	0.0436 (14)	−0.082 (2)	0.0028 (14)	−0.0279 (16)
C40	0.0638 (15)	0.098 (2)	0.0430 (12)	−0.0509 (15)	0.0085 (11)	−0.0234 (13)

### Geometric parameters (Å, °)

**Table:**

Pd1—O1^i^	1.9835 (12)	Pd2—O2	1.9826 (12)
Pd1—O1	1.9835 (12)	Pd2—O2^ii^	1.9826 (12)
Pd1—N4	2.0102 (15)	Pd2—N8^ii^	2.0136 (15)
Pd1—N4^i^	2.0102 (15)	Pd2—N8	2.0136 (15)
O1—C1	1.300 (2)	O2—C21	1.301 (2)
N1—N2	1.326 (2)	N5—N6	1.323 (2)
N1—C7	1.345 (3)	N5—C27	1.348 (3)
N2—N3	1.337 (2)	N6—N7	1.337 (2)
N2—C2	1.423 (2)	N6—C22	1.427 (2)
N3—C12	1.352 (3)	N7—C32	1.348 (3)
N4—C14	1.292 (2)	N8—C34	1.289 (2)
N4—C15	1.435 (2)	N8—C35	1.441 (2)
C1—C2	1.422 (2)	C21—C26	1.420 (2)
C1—C6	1.422 (3)	C21—C22	1.425 (2)
C2—C3	1.377 (3)	C22—C23	1.372 (3)
C3—C4	1.394 (3)	C23—C24	1.391 (3)
C3—H3B	0.9300	C23—H23A	0.9300
C4—C5	1.373 (3)	C24—C25	1.373 (3)
C4—C13	1.513 (3)	C24—C33	1.508 (3)
C5—C6	1.414 (2)	C25—C26	1.411 (3)
C5—H5A	0.9300	C25—H25A	0.9300
C6—C14	1.430 (3)	C26—C34	1.432 (3)
C7—C12	1.394 (3)	C27—C32	1.395 (3)
C7—C8	1.409 (3)	C27—C28	1.410 (3)
C8—C9	1.346 (4)	C28—C29	1.355 (4)
C8—H8A	0.9300	C28—H28A	0.9300
C9—C10	1.405 (4)	C29—C30	1.405 (5)
C9—H9A	0.9300	C29—H29A	0.9300
C10—C11	1.357 (4)	C30—C31	1.347 (4)
C10—H10A	0.9300	C30—H30A	0.9300
C11—C12	1.419 (3)	C31—C32	1.416 (3)
C11—H11A	0.9300	C31—H31A	0.9300
C13—H13A	0.9600	C33—H33A	0.9600
C13—H13B	0.9600	C33—H33B	0.9600
C13—H13C	0.9600	C33—H33C	0.9600
C14—H14A	0.9300	C34—H34A	0.9300
C15—C16	1.367 (3)	C35—C40	1.369 (3)
C15—C20	1.373 (3)	C35—C36	1.370 (3)
C16—C17	1.391 (3)	C36—C37	1.388 (3)
C16—H16A	0.9300	C36—H36A	0.9300
C17—C18	1.353 (4)	C37—C38	1.356 (4)
C17—H17A	0.9300	C37—H37A	0.9300
C18—C19	1.368 (4)	C38—C39	1.369 (4)
C18—H18A	0.9300	C38—H38A	0.9300
C19—C20	1.384 (4)	C39—C40	1.383 (3)
C19—H19A	0.9300	C39—H39A	0.9300
C20—H20A	0.9300	C40—H40A	0.9300
			
O1^i^—Pd1—O1	179.999 (1)	O2—Pd2—O2^ii^	179.999 (1)
O1^i^—Pd1—N4	89.84 (6)	O2—Pd2—N8^ii^	89.44 (6)
O1—Pd1—N4	90.16 (6)	O2^ii^—Pd2—N8^ii^	90.56 (6)
O1^i^—Pd1—N4^i^	90.16 (6)	O2—Pd2—N8	90.56 (6)
O1—Pd1—N4^i^	89.84 (6)	O2^ii^—Pd2—N8	89.44 (6)
N4—Pd1—N4^i^	180.0	N8^ii^—Pd2—N8	179.998 (1)
C1—O1—Pd1	123.88 (12)	C21—O2—Pd2	124.18 (11)
N2—N1—C7	103.01 (16)	N6—N5—C27	102.73 (17)
N1—N2—N3	116.89 (16)	N5—N6—N7	117.27 (16)
N1—N2—C2	121.96 (15)	N5—N6—C22	121.35 (15)
N3—N2—C2	121.08 (16)	N7—N6—C22	121.24 (16)
N2—N3—C12	102.22 (17)	N6—N7—C32	102.09 (17)
C14—N4—C15	118.18 (15)	C34—N8—C35	117.21 (15)
C14—N4—Pd1	122.59 (13)	C34—N8—Pd2	122.79 (12)
C15—N4—Pd1	119.21 (11)	C35—N8—Pd2	119.96 (12)
O1—C1—C2	119.72 (16)	O2—C21—C26	125.35 (16)
O1—C1—C6	124.93 (16)	O2—C21—C22	119.75 (16)
C2—C1—C6	115.34 (15)	C26—C21—C22	114.90 (16)
C3—C2—C1	121.93 (17)	C23—C22—C21	122.07 (17)
C3—C2—N2	118.38 (16)	C23—C22—N6	118.55 (17)
C1—C2—N2	119.65 (16)	C21—C22—N6	119.37 (16)
C2—C3—C4	121.97 (18)	C22—C23—C24	122.36 (18)
C2—C3—H3B	119.0	C22—C23—H23A	118.8
C4—C3—H3B	119.0	C24—C23—H23A	118.8
C5—C4—C3	117.25 (17)	C25—C24—C23	116.89 (18)
C5—C4—C13	121.85 (19)	C25—C24—C33	121.98 (19)
C3—C4—C13	120.90 (19)	C23—C24—C33	121.10 (19)
C4—C5—C6	122.23 (18)	C24—C25—C26	122.31 (18)
C4—C5—H5A	118.9	C24—C25—H25A	118.8
C6—C5—H5A	118.9	C26—C25—H25A	118.8
C5—C6—C1	120.47 (17)	C25—C26—C21	120.87 (17)
C5—C6—C14	117.37 (17)	C25—C26—C34	117.04 (17)
C1—C6—C14	122.04 (16)	C21—C26—C34	122.03 (17)
N1—C7—C12	108.81 (18)	N5—C27—C32	108.70 (19)
N1—C7—C8	129.9 (2)	N5—C27—C28	129.6 (2)
C12—C7—C8	121.2 (2)	C32—C27—C28	121.7 (2)
C9—C8—C7	117.5 (3)	C29—C28—C27	116.4 (3)
C9—C8—H8A	121.2	C29—C28—H28A	121.8
C7—C8—H8A	121.2	C27—C28—H28A	121.8
C8—C9—C10	121.7 (3)	C28—C29—C30	122.2 (3)
C8—C9—H9A	119.1	C28—C29—H29A	118.9
C10—C9—H9A	119.1	C30—C29—H29A	118.9
C11—C10—C9	122.2 (2)	C31—C30—C29	122.4 (3)
C11—C10—H10A	118.9	C31—C30—H30A	118.8
C9—C10—H10A	118.9	C29—C30—H30A	118.8
C10—C11—C12	117.1 (3)	C30—C31—C32	117.2 (3)
C10—C11—H11A	121.4	C30—C31—H31A	121.4
C12—C11—H11A	121.4	C32—C31—H31A	121.4
N3—C12—C7	109.07 (18)	N7—C32—C27	109.20 (18)
N3—C12—C11	130.8 (2)	N7—C32—C31	130.6 (2)
C7—C12—C11	120.1 (2)	C27—C32—C31	120.2 (2)
C4—C13—H13A	109.5	C24—C33—H33A	109.5
C4—C13—H13B	109.5	C24—C33—H33B	109.5
H13A—C13—H13B	109.5	H33A—C33—H33B	109.5
C4—C13—H13C	109.5	C24—C33—H33C	109.5
H13A—C13—H13C	109.5	H33A—C33—H33C	109.5
H13B—C13—H13C	109.5	H33B—C33—H33C	109.5
N4—C14—C6	126.47 (17)	N8—C34—C26	127.10 (17)
N4—C14—H14A	116.8	N8—C34—H34A	116.5
C6—C14—H14A	116.8	C26—C34—H34A	116.5
C16—C15—C20	120.1 (2)	C40—C35—C36	120.32 (19)
C16—C15—N4	121.97 (19)	C40—C35—N8	118.34 (18)
C20—C15—N4	117.94 (19)	C36—C35—N8	121.34 (18)
C15—C16—C17	119.8 (2)	C35—C36—C37	119.8 (2)
C15—C16—H16A	120.1	C35—C36—H36A	120.1
C17—C16—H16A	120.1	C37—C36—H36A	120.1
C18—C17—C16	120.2 (2)	C38—C37—C36	119.9 (2)
C18—C17—H17A	119.9	C38—C37—H37A	120.0
C16—C17—H17A	119.9	C36—C37—H37A	120.0
C17—C18—C19	120.2 (2)	C37—C38—C39	120.2 (2)
C17—C18—H18A	119.9	C37—C38—H38A	119.9
C19—C18—H18A	119.9	C39—C38—H38A	119.9
C18—C19—C20	120.3 (3)	C38—C39—C40	120.5 (3)
C18—C19—H19A	119.9	C38—C39—H39A	119.8
C20—C19—H19A	119.9	C40—C39—H39A	119.8
C15—C20—C19	119.5 (3)	C35—C40—C39	119.3 (2)
C15—C20—H20A	120.2	C35—C40—H40A	120.4
C19—C20—H20A	120.2	C39—C40—H40A	120.4
			
N4—Pd1—O1—C1	30.49 (15)	N8^ii^—Pd2—O2—C21	−151.66 (15)
N4^i^—Pd1—O1—C1	−149.51 (15)	N8—Pd2—O2—C21	28.34 (15)
C7—N1—N2—N3	1.1 (2)	C27—N5—N6—N7	1.1 (2)
C7—N1—N2—C2	178.06 (17)	C27—N5—N6—C22	176.73 (17)
N1—N2—N3—C12	−0.7 (2)	N5—N6—N7—C32	−0.9 (2)
C2—N2—N3—C12	−177.72 (18)	C22—N6—N7—C32	−176.50 (18)
O1^i^—Pd1—N4—C14	152.65 (16)	O2—Pd2—N8—C34	−24.36 (15)
O1—Pd1—N4—C14	−27.35 (16)	O2^ii^—Pd2—N8—C34	155.64 (15)
O1^i^—Pd1—N4—C15	−25.56 (14)	O2—Pd2—N8—C35	157.78 (14)
O1—Pd1—N4—C15	154.44 (14)	O2^ii^—Pd2—N8—C35	−22.22 (14)
Pd1—O1—C1—C2	163.30 (13)	Pd2—O2—C21—C26	−17.1 (3)
Pd1—O1—C1—C6	−16.5 (3)	Pd2—O2—C21—C22	163.31 (13)
O1—C1—C2—C3	−170.75 (18)	O2—C21—C22—C23	−172.27 (18)
C6—C1—C2—C3	9.1 (3)	C26—C21—C22—C23	8.1 (3)
O1—C1—C2—N2	7.0 (3)	O2—C21—C22—N6	7.4 (3)
C6—C1—C2—N2	−173.20 (16)	C26—C21—C22—N6	−172.23 (16)
N1—N2—C2—C3	−138.43 (19)	N5—N6—C22—C23	−132.8 (2)
N3—N2—C2—C3	38.4 (3)	N7—N6—C22—C23	42.7 (3)
N1—N2—C2—C1	43.8 (3)	N5—N6—C22—C21	47.5 (3)
N3—N2—C2—C1	−139.39 (19)	N7—N6—C22—C21	−136.99 (19)
C1—C2—C3—C4	−3.0 (3)	C21—C22—C23—C24	−3.6 (3)
N2—C2—C3—C4	179.28 (18)	N6—C22—C23—C24	176.75 (18)
C2—C3—C4—C5	−4.9 (3)	C22—C23—C24—C25	−3.4 (3)
C2—C3—C4—C13	175.4 (2)	C22—C23—C24—C33	178.4 (2)
C3—C4—C5—C6	6.2 (3)	C23—C24—C25—C26	5.5 (3)
C13—C4—C5—C6	−174.1 (2)	C33—C24—C25—C26	−176.4 (2)
C4—C5—C6—C1	0.2 (3)	C24—C25—C26—C21	−0.7 (3)
C4—C5—C6—C14	−175.77 (19)	C24—C25—C26—C34	−177.85 (19)
O1—C1—C6—C5	172.14 (18)	O2—C21—C26—C25	174.38 (18)
C2—C1—C6—C5	−7.7 (3)	C22—C21—C26—C25	−6.0 (3)
O1—C1—C6—C14	−12.1 (3)	O2—C21—C26—C34	−8.6 (3)
C2—C1—C6—C14	168.12 (17)	C22—C21—C26—C34	171.07 (17)
N2—N1—C7—C12	−1.0 (2)	N6—N5—C27—C32	−0.8 (2)
N2—N1—C7—C8	176.1 (2)	N6—N5—C27—C28	176.8 (2)
N1—C7—C8—C9	−176.3 (2)	N5—C27—C28—C29	−176.3 (3)
C12—C7—C8—C9	0.5 (4)	C32—C27—C28—C29	1.1 (4)
C7—C8—C9—C10	−0.9 (4)	C27—C28—C29—C30	−1.2 (5)
C8—C9—C10—C11	−0.3 (5)	C28—C29—C30—C31	0.3 (6)
C9—C10—C11—C12	1.8 (5)	C29—C30—C31—C32	0.7 (5)
N2—N3—C12—C7	0.0 (2)	N6—N7—C32—C27	0.2 (2)
N2—N3—C12—C11	−177.4 (3)	N6—N7—C32—C31	−176.7 (3)
N1—C7—C12—N3	0.6 (3)	N5—C27—C32—N7	0.4 (3)
C8—C7—C12—N3	−176.8 (2)	C28—C27—C32—N7	−177.5 (2)
N1—C7—C12—C11	178.4 (2)	N5—C27—C32—C31	177.7 (2)
C8—C7—C12—C11	1.0 (4)	C28—C27—C32—C31	−0.2 (4)
C10—C11—C12—N3	175.1 (3)	C30—C31—C32—N7	175.9 (3)
C10—C11—C12—C7	−2.1 (4)	C30—C31—C32—C27	−0.7 (4)
C15—N4—C14—C6	−171.22 (19)	C35—N8—C34—C26	−172.76 (18)
Pd1—N4—C14—C6	10.6 (3)	Pd2—N8—C34—C26	9.3 (3)
C5—C6—C14—N4	−168.91 (19)	C25—C26—C34—N8	−170.17 (19)
C1—C6—C14—N4	15.2 (3)	C21—C26—C34—N8	12.7 (3)
C14—N4—C15—C16	−60.2 (3)	C34—N8—C35—C40	122.8 (2)
Pd1—N4—C15—C16	118.12 (19)	Pd2—N8—C35—C40	−59.2 (2)
C14—N4—C15—C20	119.2 (2)	C34—N8—C35—C36	−56.0 (3)
Pd1—N4—C15—C20	−62.5 (2)	Pd2—N8—C35—C36	121.99 (18)
C20—C15—C16—C17	0.8 (4)	C40—C35—C36—C37	−0.3 (4)
N4—C15—C16—C17	−179.8 (2)	N8—C35—C36—C37	178.5 (2)
C15—C16—C17—C18	−0.7 (4)	C35—C36—C37—C38	−0.4 (4)
C16—C17—C18—C19	0.0 (4)	C36—C37—C38—C39	0.2 (4)
C17—C18—C19—C20	0.7 (5)	C37—C38—C39—C40	0.7 (5)
C16—C15—C20—C19	−0.2 (4)	C36—C35—C40—C39	1.1 (4)
N4—C15—C20—C19	−179.6 (3)	N8—C35—C40—C39	−177.7 (3)
C18—C19—C20—C15	−0.6 (5)	C38—C39—C40—C35	−1.3 (5)

Symmetry codes: (i) −*x*+1, −*y*+1, −*z*; (ii) −*x*+2, −*y*+1, −*z*+1.
